# Decade Review of Social Support in Family Gerontology: Methods, Theories, and Directions for Future Research

**DOI:** 10.1093/geroni/igaa057.1856

**Published:** 2020-12-16

**Authors:** Amber Seidel, Jeremy Yorgason, Áine Humble, Roxanne Humphris

**Affiliations:** 1 Penn State York, York, Pennsylvania, United States; 2 Brigham Young University, provo, Utah, United States; 3 Mount Saint Vincent University, Halifax, Nova Scotia, Canada

## Abstract

Social support has a rich foundation in family gerontology benefiting older adults, such as bolstering mental health, decreasing social isolation, and connecting them in families and communities. This study draws from a decade review of 995 empirical articles focusing on family gerontology from 13 of the top journals in gerontology, family, and relationships. Of the 995 articles, 86 were coded as social support or social network. Of these 86 articles, less than 3% included dyadic analyses, 95% were quantitative with just over 30% longitudinal. Over 30 different theories were identified with many building their work off multiple theories. Two key theories appear in over 30% of the articles, social support and socioemotional selectivity. Only 4% did not cite any theoretical framework. Overall, this presentation will review the methods, theories, and key findings of the social network/social support subtheme of a decade review of family gerontology and identify related gaps.

